# Swine industry stakeholders’ perception on the use of water-based foam as an emergency mass depopulation method

**DOI:** 10.1371/journal.pone.0290400

**Published:** 2023-10-20

**Authors:** Ting-Yu Cheng, Magnus R. Campler, Josie M. Rudolphi, Taylor J. Williams, Justin D. Kieffer, Steven J. Moeller, Andrew S. Bowman, Andréia G. Arruda

**Affiliations:** 1 Department of Veterinary Preventive Medicine, College of Veterinary Medicine, The Ohio State University, Columbus, Ohio, United States of America; 2 Department of Agricultural and Biological Engineering, College of Agricultural, Consumer and Environmental Sciences, University of Illinois Urbana-Champaign, Champaign, Illinois, United States of America; 3 Department of Animal Sciences, College of Food, Agricultural and Environmental Sciences, The Ohio State University, Columbus, Ohio, United States of America; UGHE: University of Global Health Equity, RWANDA

## Abstract

The U.S. pork supply chain is vulnerable to various internal and external threats and in need of prompt, comprehensive response plans. Under urgent circumstances, for example in the case of foreign disease incursions, swine farms will have to perform on-farm animal depopulation to prevent disease spread. Several animal depopulation methods including water-based foam (WBF) have been proposed and are under evaluation for feasibility in the field. However, the psychological/emotional impacts of applying depopulation methods for personnel managing and carrying on the tasks are not currently well understood. Thus, this study aimed to investigate WBF as an alternative for depopulation compared to existing methods approved by the American Veterinary Medical Association. Swine industry stakeholders were invited to voluntarily observe a WBF depopulation trial and to provide their self-reported perspectives before and after the observation. A survey was designed to explore key areas on expected and perceived method effectiveness, efficiency, and animal welfare considerations, as well as to evaluate short-term post-observation psychological impacts. Among 42 recruited stakeholders, 31.0% (13/42) were educators/researchers followed by animal health officials (26.2%, 11/42) and veterinarians (23.8%, 10/42), with an average of 11.7 ± 12.6 (n = 39) years of work experience. After the trial, respondents’ positive perception of WBF depopulation increased specifically regarding the animal loading process being less stressful than restrained in-barn depopulation options (*P* = 0.003) and by the observation of fewer swine escape attempts and vocalizations than expected (*P* < 0.001). Respondents’ positive perception of WBF also increased regarding to the time required to fill the trailer with foam, to stop hearing animal vocalization, and stop hearing animal movement, as the observed trial times were faster than their pre-observation estimates (*P* < 0.001). Additionally, 79.5% (31/39) of respondents agreed that the rapid destruction of animal populations had priority over animal welfare under urgent scenarios. Minor post-traumatic stress disorder-like (PTSD-like) symptoms from the observed trials were reported (26.7%, 4/15 respondents) one month after the observation. This study showed that the WBF depopulation process was perceived positively by swine stakeholders and may have limited short-term psychological impacts on personnel involved in animal depopulation.

## Introduction

The threat level of foreign pathogen introduction to the Unites States’ swine production supply chain has been increasing. Due to the growing global trade of swine-related products (e.g., pork products, feed ingredients, equipment, and personnel), the underlying risk for emerging disease outbreaks is increasing [1]. For instance, the imminent threat of African swine fever disease (ASV) has prompted an urgency to have response plans in place if ASV crosses onto U.S soil [2]. In addition, the aftermath of the recent supply chain disturbance including shutdown of meat processing plants during the severe acute respiratory syndrome coronavirus 2 (SARS-CoV-2) pandemic, further necessitates response plans to mitigate potential negative impacts on the U.S swine industry caused by pathogens not based in the domestic swine population [3]. During disease response situations, swine producers will likely be instructed to temporarily stop animal movements and depopulate herds to prevent disease transmission. Performing depopulation methods currently approved by the American Veterinary Medical Association (AVMA) under field conditions can be challenging from the standpoint of timeliness, economic, logistic, and animal welfare [4]. For example, depopulating large numbers of animals using physical methods including captive bolt (nonpenetrative and penetrative), gunshot, electrocution, and manual blunt force trauma can be time consuming, labor intensive, and detrimental to personnel’s mental health. Other approved methods such as anesthetic overdose, carbon dioxide stunning or movement to slaughter have the potential to be less emotionally intense but can be difficult to implement due to logistic bottlenecks caused by temporary transport restrictions or shortage of local supplies [4, 5] or limitations on carcass disposal (in the case of anesthetic overdose). Furthermore, ventilation shutdown of ventilation shutdown plus (VSD+) have been approved since 2015 by the AVMA to be used under constrained circumstances such as during infectious disease outbreaks or supply chain breakdowns during the recent SARS-Cov-2 pandemic, when slaughter or humane euthanasia was not deemed possible [5]. However, although the use of VSD/VSD+ may facilitate large-scale depopulation events and reduce workforce pressure, the methodological approach of VSD/VSD+ has raised animal welfare concerns due to the conditions and time until induced death [6–12]. The second depopulation method listed in AVMA guidelines for depopulation is the use of sodium nitrate (NaNO_2_) [4]. The administration of sodium nitrate induces methemoglobinemia due to the oxidation of ferrous ion (Fe^2+^) to ferric ion (Fe^3+^) in hemoglobin inflicting a reduction in the oxygen-carrying capacity in red blood cells leading to tissue hypoxia and death at lethal concentrations [13, 14]. The time to death after a lethal dose of NaNO_2_ in baited feral pigs have been reported to be between 120–180 minutes [13, 15] but shorter (17–101 minutes) during controlled trials using up to 3 times the lethal dose [14]. To date, NaNO_2_ has yet to be used for mass depopulation of pigs and additional research is needed to determine its efficacy during large scale implementation as well as associated welfare concerns due to the prolonged time to death.

A promising depopulation alternative for swine is the use of water-based foam (WBF), commonly used for depopulation of poultry in emergency circumstances [16]. The use of foam agents for depopulation of swine has not been investigated extensively, but the use of high expansion nitrogen gas-filled foam has recently been reported to be suitable for pigs [17, 18]. In addition, recent studies found WBF to be equally efficient in inducing cessation of movement and subsequent death in cull sows as CO_2_ or compressed nitrogen foam (CAFN_2_) [19], and that WBF could be a suitable and reliable depopulation method for pigs of different ages [20, 21]. From a personnel perspective, a possible benefit from using WBF is the reduction of both visual and handling elements during depopulation, which may help mitigate the risk of short- and long-term trauma especially during an extended depopulation scenario. Furthermore, details on the depopulation methods and plausible factors to the traumatic perceptions have not been well described, preventing a comprehensive understanding of which specific parts of the intense experience are responsible for causing the trauma. This information, if collected in a timely manner, could help justify depopulation method selection and facilitate timely interventions to prevent mental disorders in depopulation personnel.

The aim of this study was to assess the perception of swine industry stakeholders on the use of WBF in swine prior and post observation of the depopulation process in situ using a modified rendering trailer. The focus was on describing perceptions of methodology speed, likelihood of field success, swine welfare, and potential psychological and emotional impact of personnel performing the depopulation method. We hypothesized that stakeholders would perceive WBF as a better alternative for depopulation in terms of effectiveness, efficiency, and animal welfare, compared to existing approved methods following a direct observation of the process in situ. In addition, the study aimed to describe short-term psychological/emotional impacts to stakeholders after observing the WBF depopulation process.

## Materials and methods

### Study design

A survey-based study was used to explore the perceptions and perspectives of swine industry stakeholders in a mass depopulation approach using water-based foam in a modified rendering trailer. Respondents’ demographics and general perceptions of the WBF depopulation methodology were descriptively analyzed and compared before and after observing a depopulation trial. It is important to note that the original intent of the survey instruments was to capture completely de-identified data that would be internally used by the research team regarding uses of this specific method for depopulation. As such, a formal sample size was not calculated, and an IRB was only requested after-the-fact for data analysis and manuscript publication (IRB determined not necessary; Protocol no. 2021E0660 and Study Determined Exempt; Protocol no. 2023E0352).

### Trials of water-based foam (WBF) depopulation

Two similar trials were performed in two swine production sites in Ohio (Trial 1) and Indiana (Trial 2), USA, in May 2021 and May 2022, respectively, to collect research data assessing the use of WBF for swine mass depopulation (Trial 1 described in Arruda and Campler [21], Trial 2 is under peer-review at time of writing). The WBF depopulation animal trials that informed this study’s surveys were completed in accordance with animal use protocol 2020A00000036 and approved by the Institutional Animal Care and Use Committee (IACUC) at The Ohio State University. A secondary euthanasia method in the form of a penetrating captive bolt was available on site throughout both trials which would be applied by trained personnel as needed. All swine used in the studies were housed and handled according to the Guide for the Care and Use of Agricultural Animals in Research and Teaching [22].

For Trial 1, 75 cull sows (approximate purchase weight 200–275 kg) arrived two days before the trial and were assigned into three replicates, each containing 25 sows. A modified rendering trailer with a hydraulic lift system was used to load and foam animals, as described in Arruda, Campler [21]. In brief, sows were moved from the pens directly into the trailer using a loading ramp, and WBF was generated using a commercially available foam concentrate (PHOS-CHEK WD881) and a water pumping system as described in Kieffer and Campler [20]. Sows were immersed in foam and left undisturbed for 5 minutes, after which the trailer was hydraulically lifted for animals to be removed and immediately evaluated for stun efficacy and uld 5 min according to Arruda et al. 2022 (doi: 10.1111/tbed.14622) level of consciousness by several trained research team members [20]. Stakeholders were allowed to observe the process on the ground and on a scaffolding platform.

In Trial 2, 156 market-age pigs were depopulated in 12 replicates with 13 pigs each. The same trailer, depopulation procedure, and WBF generation system as described in Trial 1 were used. Upon filling the trailer with WBF, pigs were immersed for 7.5 minutes post-filling and went through the same unload process and consciousness evaluation. In this case, stakeholders were allowed to observe the process on the ground or on top of the trailer with a harness attached, as scaffolding was not available due to time and space constraints at the trial location.

### Questionnaire development

The PRE-Q, POST-Q, and FOLLOW-Q (S1–S3 Files) were constructed in consultation with an agricultural safety and mental health professional (co-author Rudolphi). Stakeholders of the swine industry were informally invited by study investigators and direct collaborators (e.g., members of state pork associations and local governmental agencies) by phone calls to observe a WBF trial and to participate in the subsequent survey. The questionnaires were designed to be a mix of multiple choice, “Yes/No” and “Select all that apply” questions to ensure ample opportunity for the respondents to provide information. An introduction differentiating animal euthanasia and depopulation based on the definition provided within the 2020 AVMA Euthanasia Guidelines [23] and 2019 Depopulation Guidelines [4] was included in the PRE-Q and POST-Q. In brief, euthanasia was defined as methods designed to “end the life of an individual animal in a way that minimizes or eliminates pain and distress”. In contrast, depopulation referred to “the rapid destruction of a population/group of animals in response to urgent circumstances with as much consideration given to the welfare of the animals as practicable”.

#### Pre-observation survey (PRE-Q)

The PRE-Q consisted of a brief introduction of the WBF depopulation process and two sections of questions on participants’ backgrounds (A. Background in S1 File) and animal welfare-related perceptions in WBF depopulation (B. Animal welfare in S1 File). The “Background” section included 17 questions regarding demographic information (e.g., years of service in the swine industry, role within the swine industry) and previous knowledge and experience in animal depopulation (e.g., awareness of AVMA guidelines, past experiences in animal euthanasia and depopulation). The “Animal Welfare” section consisted of 13 questions regarding how participants perceived WBF depopulation in comparison to other AVMA-approved methods in terms of addressing multiple animals at one time, animal stress, and logistical effectiveness. In addition, participants were asked to predict the amount of escape attempts, distress-induced animal vocalizations, and the level of animal suffering they would observe during the WBF depopulation process. Finally, the animal welfare section included questions that allowed respondents to predict efficiency and effectiveness of the process, including time to immerse all animals in a trailer with WBF, time to stop hearing animal vocalizations, time to stop hearing animal movements, and the proportion of animals that would be deemed unconscious at the end of the process.

#### Post-observation survey (POST-Q)

The POST-Q was designed to compare animal welfare perceptions (A. Animal Welfare in S2 File) and efficiency and effectiveness estimates (B. Methodology Speed in S2 File) with PRE-Q, and further investigate participants’ perspectives of field feasibility (C. Field Condition Success in S2 File) and personnel-related safety and health (D. Direct and Indirect Staff Health and Safety in S2 File) after observing the WBF depopulation process. For section B, although timers and stopwatches were not provided, participants were allowed to estimate using their personal devices. The “Animal Welfare” section was structured in 14 questions, similar to the corresponding section in PRE-Q, for comparisons purposes. Participants’ perceptions regarding field feasibility (e.g., probability to be implemented in the field, application to animals with different ages) and ranking compared to AVMA-approved in-barn methods (e.g., blunt force trauma, non-penetrative/penetrative captive bolts, ventilation shutdown, CO_2_ chamber, electrocution) were collected by eight questions in the “Field Condition Success” section. The “Direct and Indirect Staff Health and Safety” section consisted of a brief statement of the non-toxicity of the foaming agent and 10 questions probing participants’ perceptions on their previous euthanasia and depopulation experiences and from the observation of the WBF depopulation process.

#### Follow-up questionnaire (FOLLOW-Q)

The FOLLOW-Q (S3 File) consisted of seven questions to assess the perceived short-term impacts and effects by the observation of WBF depopulation process. Participants were asked to rank the level of their perceptions/experiences (from “Not at all” to “Extremely”) on certain negative signs (e.g., disturbing thoughts, upsetting recollections) that may be linked to the observation of WBF depopulation process, and to rank the severity of any experienced signs. These questions were adapted from the validated Posttraumatic Stress Disorder Checklist (PCL-6) [24].

### Statistical analysis

Surveys were manually entered into an Excel database (Microsoft Corp^®^, Redmond, WA). Descriptive analysis was performed to describe demographics, including professional role and years of service in the swine industry. The frequency and percentage of responses were reported among categories for nominal questions, while numeric responses were summarized as means with standard deviations (SD). Where a range of numbers was provided, e.g., 30–50% of pigs, the upper bound of the ranges was used for analysis. Responses to multiple choice questions paired in PRE-Q and POST-Q (e.g., ensuring efficient depopulation, stress of animal loading, number of escape attempts) were compared using cumulative link mixed models (i.e., effects of the observation on stakeholders’ responses). Models were constructed by using the ordinal responses to questions paired in PRE-Q and POST-Q (Table 1) as the outcomes, observation status (pre/post) and trial (1/2) as fixed effects, and survey ID (anonymous participant identifier) as a random effect. Stakeholders’ PRE-Q predictions and POST-Q estimates of time to trailer fill, time to hearing the last animal movement, and percentage of unconscious animals were compared using a non-parametric paired Wilcoxon signed-rank test. Post-Q estimates were further compared with the exact average time to fill the trailer and to the animals’ cessation of movements using the one-sample Wilcoxon signed-rank test. In addition, the association between participants’ observation locations and their post-observation responses (Table 1) was investigated using a non-parametric Kruskal-Wallis one-way analysis of variance (ANOVA) for numerical predictions/estimates (e.g., time to trailer fill, time to last animal movement) and Fisher’s Exact Test for ordinal responses (e.g., ensuring efficient depopulation, number of escape attempts). Responses to the short-term impact assessment (FOLLOW-Q) were descriptively analyzed. Statistical significance was declared at *P* < 0.05; and all analysis and graphs were conducted using R 4.0.4 [25].

**Table 1:** Swine industry stakeholders’ perception regarding logistic, speed, efficiency, and animal welfare of swine depopulation using the water-based foam before and after observing two field trials.

			Response	
Question			Pre-observation	Post-observation
PRE-Q: The ability to address multiple animals simultaneously using foaming is beneficial to ensure efficient depopulation.	Number of responses		41	39
	Strongly agree		20 (48.8%)	21 (53.8%)
	Agree		19 (46.3%)	17 (43.6%)
POST-Q: The possibility to destroy multiple animals simultaneously using foaming is beneficial to ensure good depopulation.	Neither agree nor disagree		2 (4.9%)	1 (2.6%)
	Disagree		0 (0.0%)	0 (0.0%)
	Strongly disagree		0 (0.0%)	0 (0.0%)
PRE-Q: The process of moving animals into a foaming trailer is less stressful to the animal compared to in-barn based methods.	Number of responses		41	39
	Strongly agree		2 (4.9%)	4 (10.3%)
	Agree		6 (14.6%)	14 (35.9%)
POST-Q: The process of moving animals into a foaming trailer is less stressful to the animal compared to in-barn based methods (inside the facilities).				
	Neither agree nor disagree		20 (48.8%)	16 (41.0%)
	Disagree		13 (31.7%)	5 (12.8%)
	Strongly disagree		0 (0.0%)	0 (0.0%)
PRE-Q: How many escape attempts do you anticipate observing during the trial (animal is trying to get head above foam, jumping or thrusting body against wall to escape the foam, standing on hind legs against wall to escape the foam)?	Number of responses		41	39
	One or less		12 (29.3%)	29 (74.4%)
	2–5		15 (36.6%)	10 (25.6%)
POST-Q: How many escape attempts did you see/notice during the trial (jumping or thrusting body against wall)	6–10		11 (26.8%)	0 (0.0%)
	> 10		3 (7.3%)	0 (0.0%)
PRE-Q: How much non-normal vocalization (squeals, sounds indicative of excess stress/fear/pain) do you expect to hear during the foaming process (across the group of animals)?	Number of responses		41	39
	None		2 (4.9%)	28 (71.8%)
	Some (1–5)		29 (70.7%)	11 (28.2%)
POST-Q: How much vocalization did you hear during the trial?				
	Frequent (> 5)		10 (24.4%)	0 (0.0%)
PRE-Q: How long (approximately, in minutes) do you believe it will take to fill the trailer/container completely with foam?	Number of responses		41	37
	Time (minute)		8.5 ± 7.4	1.5 ± 1.0
POST-Q: How long (approximately, in minutes) did it take to fill the container completely with foam?				
PRE-Q: How long (approximately, in minutes) do you believe it will take for you to stop hearing animal vocalization?	Number of responses		41	32
	Time (minute)		7.1 ± 5.7	1.3 ± 3.6
POST-Q: How long (approximately, in minutes) did it take for you to stop hearing animal vocalization?				
PRE-Q: How long (approximately, in minutes) do you believe it will take for you to stop hearing animal movement?	Number of responses		41	29
	Time (minute)		8.4 ± 6.2	2.3 ± 0.9
POST-Q: How long (approximately, in minutes) did it take for you to stop hearing animal movement?				
PRE-Q: What percentage of animals in the trial do you believe will be deemed unconscious after the pre-defined trial period?	Number of responses		40	37
	Average ± standard deviation		94.5% ± 7.4	99.5% ± 3.3
POST-Q: What percentage of animals in the trial were unconscious after the foaming procedure was complete?	Percentile	25	90.0%	100.0%
		50	98.5%	100.0%
		75	100.0%	100.0%

## Results

For Trial 1, the mean (±standard deviation) time (in mm:ss) to fill the trailer with WBF was 01:23 ± 00:12 and with the time to cessation of pig movements 02:11 ± 00:35 [21]. For Trial 2, the average time to fill the trailer with WBF was 01:27 ± 01:12 and the time to the cessation of swine movement was 02:46 ± 00:42. Across two trials, the trailer was filled in approximately 01:25 and the animals stopped moving at approximately 02:29. Vocalization was not identified during neither trial.

### Pre-observation questionnaire (PRE-Q)

Overall, 33 and 9 animal stakeholders observed WBF trials 1 and 2, respectively, and returned responses, for a total of 42 surveys. Respondents could select multiple roles and duties that applied, and the highest proportion identified themselves as educators/researchers (31.0%, 13/42) followed by animal health officials (26.2%, 11/42), veterinarians (23.8%, 10/42), Others (e.g., veterinary students, national organization representatives, administrative personnel) (19.0%, 8/42), animal welfare professionals (4.8%, 2/42), and farm owner (2.4%, 1/42), with an average of 11.7 ± 12.6 (n = 39) years of work experience. Close to three-quarters (73.8%, 31/42) of the respondents were aware and knowledgeable of the AVMA depopulation guidelines and approximately half (48.7%, 19/39) had consulted this resource during a depopulation scenario. Penetrating captive bolt was reported as the most observed euthanasia method (60.0%, 24/40) followed by gunshot (57.5%, 23/40) and carbon dioxide inhalation (50.0%, 20/40). Furthermore, 35.9% (14/39) of respondents had experience with actively performing or assisting during a depopulation scenario and 22.5% (9/40) of the respondents had experience observing WBF depopulation in the past.

Prior to the trial, most respondents (95.1%, 39/41) considered WBF better (“Agree” and “Strongly agree”) than existing euthanasia and depopulation methods in warranting efficient depopulation. Respondents showed concerns in introducing additional stress through the animal loading process (31.7% disagreed, 13/41), but overall agreed (55.0% agreed, 22/40) it was more logistically effective compared to in-barn depopulation methods. The majority (92.7%, 38/41) of respondents expected to see less than 10 escape attempts, at least one distress-induced vocalization (95.1%, 39/41), and little to some suffering (92.5%, 37/40) during the WBF depopulation process. On average, respondents predicted an 8.5-minute time frame from start to finish of the foaming procedure, including fill time of the trailer (8.5 ± 7.4 minutes, n = 41), cessation of animal vocalization (7.1 ± 5.7 minutes, n = 41), and cessation of animal movements (8.4 ± 6.2 minutes, n = 41) (Table 1). In addition, 50.0% of the respondents expected that over 98.0% of animals would be unconscious by the end of foaming process (Table 1).

### Post-observation questionnaire (POST-Q)

For Trial 1, 30 survey respondents overlooked the trailer from the scaffolding (n = 18), on top of the trailer (n = 5), and on the ground beside the trailer (n = 7). Among 9 respondents in Trial 2, all participants observed the process at least once from the top of the trailer and 5 also observed it on the ground by the trailer. After the observation, although similar perceptions were reported on the efficiency of addressing multiple animals (*P* = 0.528), respondents (n = 39) were more positive on the WBF depopulation by considering the animal loading less stressful than restrained in-barn depopulation options (*P* = 0.003) and observing less escape attempts and vocalizations (*P* < 0.001). Based on respondents’ estimates (n = 29), animal movement ceased within 2.3 ± 0.9 minutes after the trailer was filled with WBF. In addition, respondents (79.5%, 31/39) agreed that the rapid destruction of animal populations had the priority over animal welfare under urgent scenarios. Compared to the estimated predictions in PRE-Q (Table 1), respondents reported shorter periods of time to fill the trailer with WBF (PRE-Q versus POST-Q; 8.5 ± 7.4 versus 1.5 ± 1.0 minutes, *P* < 0.001), to stop hearing animal vocalization (7.1 ± 5.7 versus 1.3 ± 3.6 minutes, *P* < 0.001), and to stop hearing animal movement (8.4 ± 6.2 versus 2.3 ± 0.9 minutes, *P* < 0.001). Respondents’ POST-Q estimates were similar to the actual time to fill the trailer (mean = 01:25, *P* = 0.65), but longer than the actual time to the cessation of animal movements (mean = 02:29, *P* = 0.04). In addition, a higher percentage of unconscious animals was reported compared to the PRE-Q estimated predictions (94.5% ± 7.4 versus 99.5% ± 3.3; *P* < 0.001). Participants’ post-observation estimated predictions and responses were not dependent on their trial observation locations (*P* > 0.05).

Overall, most respondents (84.6%– 100% selected “Very good” or “Good”) perceived WBF to be a good swine depopulation approach considering the process from loading to unloading, speed, swine welfare, and personnel safety. In addition, 76.9% (30/39) of respondents did not find the procedure to be unpleasant to observe, but nearly half (45.5%, 15/33) were concerned that WBF depopulation would require more equipment and resources compared to the use of captive bolts. In addition, 41.0% (16/39) of respondents perceived WBF to be equal or worse regarding biosecurity issues compared to currently available depopulation methods.

In respect to personnel’s safety and health, most respondents (72.2%, 26/36) reported occasionally recalling previous euthanasia events, while 42.1% (16/38) believed the WBF depopulation process could be a concern in staff and practitioners’ mental fortitude. Nevertheless, most respondents did not experience stress (92.3%, 36/39, selected “Not at all”), upset (82.1%, 32/39), aversion (100.0%, 39/39), irritation (97.4%, 38/39), anxiousness (97.4%, 38/39) immediately after the observation.

### Follow-up questionnaire (FOLLOW-Q)

Among 41 stakeholders that returned both PRE-Q and POST-Q, 15 responded to the FOLLOW-Q one-month post-trial (response rate: 38.5%). Overall, stakeholders experienced none to minor post-traumatic stress disorder-like (PTSD-like) symptoms from the WBF swine depopulation trials that they observed (Table 2). Two respondents reported “a little” for the frequency of repeated, disturbing memories, thoughts, or images from the depopulation trials, and one experienced being upset upon being reminded about the trails. In addition, one respondent reported having difficulty in concentration.

**Table 2:** Short-term mental effects on participated swine industry stakeholders one month after observing two water-based foam swine depopulation trials.

Question		Response
Since you participated in the foaming depopulation trial a few weeks ago, have you experienced repeated, disturbing memories, thoughts, or images from the depopulation trial?	Number of responses	15
	Not at all	13 (86.6%)
	A little bit	2 (13.3%)
	Moderately	0 (0.0%)
	Quite a bit	0 (0.0%)
	Extremely	0 (0.0%)
Since you participated in the foaming depopulation trial a few weeks ago, have you experienced feeling very upset when something reminded you about the depopulation trial?	Number of responses	15
	Not at all	14 (93.3%)
	A little bit	1 (6.7%)
	Moderately	0 (0.0%)
	Quite a bit	0 (0.0%)
	Extremely	0 (0.0%)
Since you participated in the foaming depopulation trial a few weeks ago, have you avoided activities or situations because they reminded you about the foaming trial?	Number of responses	15
	Not at all	15 (100.0%)
	A little bit	0 (0.0%)
	Moderately	0 (0.0%)
	Quite a bit	0 (0.0%)
	Extremely	0 (0.0%)
Since you participated in the foaming depopulation trial a few weeks ago, have you felt irritable or had angry outbursts?	Number of responses	15
	Not at all	15 (100.0%)
	A little bit	0 (0.0%)
	Moderately	0 (0.0%)
	Quite a bit	0 (0.0%)
	Extremely	0 (0.0%)
Since you participated in the foaming depopulation trial a few weeks ago, have you had difficulty concentrating?	Number of responses	15
	Not at all	14 (93.3%)
	A little bit	1 (6.7%)
	Moderately	0 (0.0%)
	Quite a bit	0 (0.0%)
	Extremely	0 (0.0%)
Since you participated in the foaming depopulation trial a few weeks ago, have you felt jumpy or easily startled?	Number of responses	15
	Not at all	15 (100.0%)
	A little bit	0 (0.0%)
	Moderately	0 (0.0%)
	Quite a bit	0 (0.0%)
	Extremely	0 (0.0%)

## Discussion

This manuscript adds to the body of knowledge on an important topic in veterinary medicine; the personnel perceptions regarding a novel WBF depopulation method for emergency situations. Social impacts to personnel that actively participates in and/or observes mass depopulation events should not be overlooked [26, 27]. These refer to indirect health consequences at the individual, family, or community level that could involve a wide range of swine stakeholders such as veterinarians, farm owners and animal caretakers, and first responders. Prolonged exposure to euthanasia or depopulation practices is known to incur both mental and physical fatigue [26, 28, 29], which may also increase risks of injury for both personnel and swine during animal handling and restraint, application of the depopulation methods, or due to improper equipment operation [4, 30]. Detrimental psychological and emotional impacts have been reported in field personnel during the eradication events of Foot and Mouth Disease in The Netherlands, the United Kingdom, and Japan, as well as eradication of bovine spongiform encephalopathy in Canada [27, 31, 32]. Of note, most of these publications reflect either expert opinion or perceptions on a method’s impact on personnel well-being [33] or assessments of post-traumatic stress symptoms conducted several years after the disasters [27, 31, 32], instead of directly measuring the impact. Furthermore, details on the depopulation methods and plausible factors to the traumatic perceptions were not described, preventing a comprehensive understanding of which specific parts of the intense experience are responsible for causing the trauma. This information, if collected in a timely manner, could help justify depopulation method selection and facilitate timely interventions to prevent mental disorders in depopulation personnel.

Given the majority of respondents in our survey were educators, animal officials, and veterinarians, the moderate knowledge level and reference to the AVMA depopulation guidelines [4] was unexpected since they were likely trained by formal education programs and courses. This highlights the need for education on the existence of guidelines specifically for depopulation of animals. Such efforts have been conducted for euthanasia, so a similar model could be developed in future efforts [34–37].

After observing the trials, respondents became more optimistic in terms of efficiency and animal welfare of using the WBF for depopulation purposes when compared to existing approved methods, in contrast to their initial assessment. In addition, after observing the WBF trials, respondents perceived the logistics of moving and loading animals into the trailer to be more logistically efficient compared to logistics from currently approved depopulation methods. Respondents also became less concerned about potential additional stress to animals during the loading process compared to their initial expectations prior to observing the trials. Furthermore, respondents expected a higher number of animal escape attempts, animal vocalizations and a longer conscious time of potential suffering before death was induced, compared to their experience during the depopulation trial. In addition, the observed and reported time to trailer foam fill, cessation of vocalization, and cessation of movement were all significantly shorter than respondents’ pre-observation predictions. Although some participants reported concerns about resource accessibility for WBF depopulation when compared to captive bolt stunning and gunshot, respondents overall measured WBF as a better depopulation approach when it came to logistics, animal welfare, speed, personnel safety, and aesthetics.

Regarding personnel mental health, over 95% of respondents did not report common post-traumatic symptoms (irritation, distraction, and anxiety) after the observation, and less than half (42.1%) concerned WBF depopulation would weaken the mental fortitude of depopulation staff and caretakers. Minor short-term PTSD-like symptoms such as negative thoughts, flashback images, feeling upset and having difficulty concentrating were reported by four respondents. Performing animal euthanasia and depopulation can be a moral and ethical burden and introduce traumatic stress to practitioners and those involved with the activity [38, 39]. Symptoms of perpetration-induced traumatic stress, a consequence of actively participating in killing of healthy animals, including nightmares, intrusive memories, and avoidance, has been reported in farm caretakers that euthanized animals [29, 31, 40–42]. A recent study reported farm frontline culling workers (n = 200; 70.0% had worked with chicken, 53.0% with cows, 37.5% with pigs) suffered intensive guilt, distress, and irritation, and varied PTSD symptoms [43] that could be triggered by sights, sounds, and smells [44]. Although few respondents reported PTSD symptoms in this study, additional research is needed across a larger sample size and over a longer timeframe before any conclusive inferences regarding the impact of WBF on personnel can be drawn.

This study also comes with limitations. First, the findings of this study should be interpreted and generalized considering the small sample size and the fact that we took advantage of other on-going studies for this work. Although both trials were performed in May, animals and the depopulation process may be affected by the weather given two animal trials were performed at different locations with one year interval. In addition, since respondents were recruited on a voluntary basis, they may already be interested and willingly to observe the depopulation trials prior to the recruitment and could be more resilient to consequential negative perceptions. Furthermore, study results may also have limited extrapolation to different personnel populations given the limited inclusion of on-farm personnel (e.g., farm owner, farm staff/caretaker, truck driver). Although the top three recruited stakeholders (educator/researchers, animal health officials, veterinarians) will be involved in animal depopulation events, they are likely to play a role as a supervisor rather than practitioner. Farm personnel, on the other hand, may not have the opportunity to refrain from the depopulation process under urgent circumstances and may be more likely to develop aversive attitudes. The follow-up questionnaire response rate of 38.5% was low on an already limited sample size, but in line with previous findings on average response rates (34% - 41%) for online surveys/questionnaires [45–47]. Thus, future studies should aim for larger sample sizes and to investigate the perspective of farm personnel, and focus on describing and exploring both short- and long-term psychological impacts of WBF and to evaluate mental health using additional validated PTSD assessment instruments (e.g., 30-item Clinician-Administered PTSD Scale) [48]. They should also attempt to obtain qualitative data, for example using semi-structured interviews, which would likely provide richer and more detailed feedback. Nevertheless, this study provides an overall supportive sentiment for the field application of WBF depopulation from swine industry stakeholders and limited short-term personnel mental health implications.

## Supporting information

S1 File Survey participants were requested to complete the pre-observation questionnaire prior to the observation of water-based foam swine depopulation trials. (PDF)

S2 File Survey participants were requested to complete the post-observation questionnaire after the observation of water-based foam swine depopulation trials. (PDF)

S3 File Survey participants were requested to complete the follow-up questionnaire one month after the observation of water-based foam swine depopulation trials. (PDF)

S1 Data (XLSX)
