# Supplementary material for: Swine industry stakeholders’ perception on the use of water-based foam as an emergency mass depopulation method
Source: PLoS One. 2023 Oct 20;18(10):e0290400. doi: 10.1371/journal.pone.0290400 (PMC10588842; doi:10.1371/journal.pone.0290400)
Supplement: S1 File — (PDF) [file pone.0290400.s001.pdf]

Questionnaire Number: PRE – 001 Date:

Type: H_2O-based foam Animal species: Number of animals in the trial:

We appreciate your time and help on this project. Your answers will be kept confidential. Your responses will help to improve swine depopulation methodology that may be used in the presence of a disease event, unexpected environmental condition, or in response to emergency situations such as experienced during the COVID-19 pandemic.

PLEASE READ BEFORE CONTINUING.

Euthanasia is a methodology designed to ensure a **humane**, painless, and quick death under standard production and environmental conditions. In contrast, **depopulation** refers to the rapid destruction of a population/group of animals in response to urgent circumstances with as much **consideration** given to the welfare of the animals as practicable. Depopulation strategies aim to recover and harvest as many of the affected animals as possible, but may result in waste of animal products based on the capacity of the market supply chain and the method.

The application of a foaming agent involves rapid, full coverage of animals with foam within a fully contained enclosure (depopulation trailer, depopulation containment vessel). Following full body foam coverage, the process results in rapid asphyxiation and loss of consciousness, followed by death due to the displacement of oxygen. Foaming is being considered as an option for depopulation of groups of animals under urgent circumstances.

The trailer you are viewing is purpose built for delivery of either gaseous or foam-based agents being considered for depopulation of animals (livestock and poultry) in groups. The self-contained trailer will allow containment of animals prior to application of the depopulation process and allow containment of animal carcasses and materials to a time of disposal at an approved location.

A. BACKGROUND

1. What option best describes your current title/role (please select primary role)?

☐ Veterinarian ☐ Farm owner ☐ Farm manager

☐ Feed mill employee ☐ Farm staff/caretaker ☐ Transporter/driver

☐ Animal health official (USDA, Regulatory body) ☐ Animal welfare professional

☐ Educator/ Researcher ☐ Other (please specify):________________________________

2. How many years have you been working within this production industry?_____ years

3. Are you aware of the AVMA (American Veterinary Medical Association) Guidelines for the Depopulation of Animals?

☐ Yes ☐ No

4. **Would you consider yourself knowledgeable regarding the AVMA (American Veterinary Medical Association) Guidelines for the Depopulation of Animals?** ☐ Yes ☐ No
5. **Have you ever referred to the AVMA (American Veterinary Medical Association) Guidelines for the Depopulation of Animals in a depopulation scenario?** ☐ Yes ☐ No
6. **Have you ever personally performed, participated in, or observed animal euthanasia?**

   ☐ Yes- which specie(s)? ____☐ No

7. **Please select method(s) of euthanasia that you have <u>observed.</u> (Select all that apply)**

   ☐ Blunt force trauma ☐ Non-penetrating captive bolt ☐ Penetrating captive bolt

   ☐ Electrocution ☐ Carbon dioxide (CO_2) ☐ Gunshot

   ☐ Other (please specify):______________________________ ☐ None

8. **Please select method(s) of euthanasia that you have <u>conducted?</u> (Select all that apply)**

   ☐ Blunt force trauma ☐ Non-penetrating captive bolt ☐ Penetrating captive bolt

   ☐ Electrocution ☐ Carbon dioxide (CO_2) ☐ Gunshot

   ☐ Other (please specify):_____________________________ ☐ None

9. **In your current role, have you been/are you actively involved in euthanasia decisions for <u>the animal species being utilized in today's scenario?</u>** ☐ Yes ☐ No
10. **Please select the level of training you have received for euthanasia for the species observed in the present scenario.**

    ☐ Trained by farm personnel (colleagues, supervisors, vets, shadowing, practice under supervision)

    ☐ Trained by third-party educators (e.g. National Pork Board Program(s))

    ☐ Trained in formal education programs (high school, apprenticeship school, college/university)

    ☐ Topic-specific higher education course work (e.g. MS, PhD, Vet Med training)

    ☐ Other (please specify): ________________________________ ☐ None

11. **How many years do you have of overall experience with euthanasia for <u>the species used in today's scenario</u>? _____________ years**
12. **How frequently do you personally conduct euthanasia?**

☐ Daily ☐ Weekly ☐ Bi-weekly ☐ Monthly ☐ Other:___________________

13. For the species you are observing in today's scenario, which <u>euthanasia</u> method(s) do you perform and how often (please consider last 1-5 years)?

	Daily	Weekly	Every other Week	Monthly	Less frequent	N/A
Blunt force trauma	☐	☐	☐	☐	☐	☐
Non-penetrating captive bolt	☐	☐	☐	☐	☐	☐
Penetrating captive bolt	☐	☐	☐	☐	☐	☐
Electrocution	☐	☐	☐	☐	☐	☐
Gunshot	☐	☐	☐	☐	☐	☐
Gas (CO_2)	☐	☐	☐	☐	☐	☐
Other (specify):______________	☐	☐	☐	☐	☐	☐

13. Have you ever performed/ helped with depopulation efforts? ☐ Yes ☐ No

14. For the species you are observing in today's scenario, which <u>depopulation</u> method(s) do you perform and how often (please consider last 1-5 years)?

	Daily	Weekly	Every other Week	Monthly	Less frequent	N/A
Blunt force trauma	☐	☐	☐	☐	☐	☐
Non-penetrating captive bolt	☐	☐	☐	☐	☐	☐
Penetrating captive bolt	☐	☐	☐	☐	☐	☐
Electrocution	☐	☐	☐	☐	☐	☐
Gunshot	☐	☐	☐	☐	☐	☐
Gas (CO_2)	☐	☐	☐	☐	☐	☐
Ventilation Shut Down (alone or Plus)	☐	☐	☐	☐	☐	☐
Carbon Monoxide	☐	☐	☐	☐	☐	☐
Other (specify):______________	☐	☐	☐	☐	☐	☐

15. Do you have personal experience (actively participated, observed, facilitated) from last years (2020) swine depopulation due to the COVID-19 pandemic specifically? ☐ Yes ☐ No

16. Have you ever observed foaming methodology for the purposes of euthanasia or depopulation purposes in person? If so, in which species and where (please provide species, country or state)

◻Yes (not in the U.S): Species:__________________ Country: _______________

◻ Yes (in the U.S) Species:__________________ State: __________________

◻ No

B. ANIMAL WELFARE

Based on the information provided, please respond to the following questions.

1. **On paper, foaming sounds better compared to existing euthanasia methods.**

☐ Strongly Agree ☐ Agree ☐Neither agree nor disagree ☐ Disagree ☐Strongly disagree

2. **On paper, foaming sounds better compared to existing depopulation methods.**

☐ Strongly Agree ☐ Agree ☐Neither agree nor disagree ☐ Disagree ☐Strongly disagree

3. **The ability to address multiple animals simultaneously using foaming is beneficial to ensure efficient depopulation.**

☐ Strongly Agree ☐ Agree ☐ Neither agree nor disagree ☐ Disagree ☐ Strongly disagree

4. **The process of moving animals into a foaming trailer is less stressful to the animal compared to in-barn based methods.**

☐ Strongly Agree ☐ Agree ☐ Neither agree nor disagree ☐ Disagree ☐ Strongly disagree

5. **The process of moving animals into a foaming trailer is more effective logistically compared to in-barn based methods.**

☐ Strongly Agree ☐ Agree ☐ Neither agree nor disagree ☐ Disagree ☐ Strongly disagree

6. **How many escape attempts do you anticipate to observe during the trial (animal is trying to get head above foam, jumping or thrusting body against wall to escape the foam, standing on hind legs against wall to escape the foam)?**

☐ One or less ☐ 2 to 5 ☐ 6 to 10 ☐ >10

7. **How much non-normal vocalization (squeals, sounds indicative of excess stress/fear/pain) do you expect to hear during the foaming process (across the group of animals)?**

   ☐ None ☐ Some (1 to 5) ☐ Frequent (>5)

8. **How much animal suffering (perceived pain or distress in the animal) would you expect to see during this foaming depopulation trial?**

None	Little	Some	Substantial
☐	☐	☐	☐

9. **How much animal suffering (perceived pain or distress in the animal) would you expect with the following depopulation methods?**

	None	Little	Some	Subtantial
Blunt force trauma	☐	☐	☐	☐
Non-penetrating captive bolt	☐	☐	☐	☐
Penetrating captive bolt	☐	☐	☐	☐
Electrocution	☐	☐	☐	☐
Gunshot	☐	☐	☐	☐
Gas (Carbon Dioxide, $CO2$)	☐	☐	☐	☐

METHODOLOGY SPEED

1. **How long (approximately, in minutes) do you believe it will take to <u>fill the trailer/container</u> completely with foam? ________________ minutes**
2. **How long (approximately, in minutes) do you believe it will take for you to <u>stop hearing animal vocalization?</u> __________________ minutes**
3. **How long (approximately, in minutes) do you believe it will take for you to <u>stop hearing animal movement?</u> __________________ minutes**
4. **What percentage of animals in the trial do you believe will be deemed <u>unconscious</u> after the pre-defined trial period? __________ %**

Please feel free to share other comments or concerns:

THANK YOU for your participation!
