# Supplementary material for: Swine industry stakeholders’ perception on the use of water-based foam as an emergency mass depopulation method
Source: PLoS One. 2023 Oct 20;18(10):e0290400. doi: 10.1371/journal.pone.0290400 (PMC10588842; doi:10.1371/journal.pone.0290400)
Supplement: S2 File — (PDF) [file pone.0290400.s002.pdf]

Questionnaire Number: POST – 001 Date:

Type: H2O-based foam Animal species: Number of animals in the trial:

Thank you for taking the time to participate in this post-trial questionnaire. Before you start, please take a minute to refresh your memory regarding the distinction between euthanasia and depopulation.

Euthanasia is a methodology designed to ensure a **humane**, painless, and quick death under standard production and environmental conditions. In contrast, **depopulation** refers to the rapid destruction of a population/group of animals in response to urgent circumstances with as much **consideration** given to the welfare of the animals as practicable. Depopulation strategies aim to recover and harvest as many of the affected animals as possible, but may result in waste of animal products based on the capacity of the market supply chain.

A. ANIMAL WELFARE

1. What was your observation location in relation of the foaming trailer?

☐ On top of trailer ☐ Level with trailer roof ☐ On ground beside trailer

☐ Other (please specify):_________________________

2. After seeing the foaming demonstration, in your opinion, this method is better compared to existing approved DEPOPULATION methods (timely death with humane considerations given). Please refer to the separate depopulation fact sheet if need be.

☐ Strongly Agree ☐ Agree ☐ Neither agree nor disagree ☐ Disagree ☐ Strongly disagree

3. The possibility to destroy multiple animals simultaneously using foaming is beneficial to ensure good depopulation.

☐ Strongly Agree ☐ Agree ☐ Neither agree nor disagree ☐ Disagree ☐ Strongly disagree

4. The process of moving animals into a foaming trailer is less stressful to the animal compared to on-farm-based depopulation (inside the facilities).

☐ Strongly Agree ☐ Agree ☐ Neither agree nor disagree ☐ Disagree ☐ Strongly disagree

5. The foaming method ensures that good animal welfare standards are fulfilled.

☐ Strongly Agree ☐ Agree ☐ Neither agree nor disagree ☐ Disagree ☐ Strongly disagree

6. How long did it take for animals to cease movement after the container was filled with foam? _____________ minutes

7. How many escape attempts did you see/notice during the trial (jumping or thrusting body against wall)

☐ One or less ☐ 2 to 5 ☐ 6 to 10 ☐ >10

8. How much vocalization did you hear during the trial?

☐ None ☐ Some (1 to 5) ☐ Frequent (>5)

9. How much animal suffering (pain, distress) did you see during the trial?

☐ None ☐ Some ☐ A lot

10. In your opinion, does the rapid destruction of a population of animals <u>in a time of emergency</u> outweighs the loosening of the animal welfare regulations to ensure a humane, painless and quick death (Euthanasia)?

☐ Yes ☐ No ☐ Unsure

B. METHODOLOGY SPEED

1. **How long (approximately, in minutes) did it take to <u>fill the container</u> completely with foam? ____________ minutes**
2. **How long (approximately, in minutes) did it take for you to stop hearing <u>animal vocalization</u>? __________________ minutes**
3. **How long (approximately, in minutes) did it take for you to stop hearing <u>animal movement</u>? ____________ minutes**
4. **What percentage of animals in the trial were <u>unconscious</u> after the foaming procedure was complete? ______________________ %**

C. FIELD CONDITION SUCCESS

1. **What would you say is the probability that this method would succeed under field conditions, or be implemented on an industrial scale, on a scale from 0 to 100%? ______ %**
2. **Do you believe the method can be used on pigs of different ages to the ones you saw today?**

☐ Yes ☐ No (please clarify):____

3. **How would you rank each of the individual parts of the foaming depopulation method?**

	Very Poor	Poor	Fair	Good	Very Good
Loading	☐	☐	☐	☐	☐
Filling the container with foam	☐	☐	☐	☐	☐
Successfully submerging animals in foam	☐	☐	☐	☐	☐
Opening the container to assess unconsciousness/ death	☐	☐	☐	☐	☐
**Overall Logistics**	☐	☐	☐	☐	☐
Animal welfare	☐	☐	☐	☐	☐

Speed	☐	☐	☐	☐	☐
Personnel safety	☐	☐	☐	☐	☐
Required resources	☐	☐	☐	☐	☐
Aesthetics (unpleasant to observe)	☐	☐	☐	☐	☐
**Overall Method**	☐	☐	☐	☐	☐

4. Compared to <u>gas-based Carbon-Dioxide (CO_2)</u> methodology for depopulation purposes, and considering the method as a whole, how does water-based foaming rank in your opinion?

	Worse	Same	Better
Overall method	☐	☐	☐
Logistics	☐	☐	☐
Animal welfare	☐	☐	☐
Speed	☐	☐	☐
Personnel safety	☐	☐	☐
Required resources	☐	☐	☐
Aesthetics (unpleasant to observe)	☐	☐	☐
No opinion/Not qualified to judge	☐		

5. Compared to <u>penetrating or non-penetrating captive bolt</u> methodology for depopulation purposes, and considering the method as a whole, how does water-based foaming rank in your opinion?

	Worse	Same	Better
Overall method	☐	☐	☐
Logistics	☐	☐	☐
Animal welfare	☐	☐	☐
Speed	☐	☐	☐
Personnel safety	☐	☐	☐
Required resources	☐	☐	☐
Aesthetics (unpleasant to observe)	☐	☐	☐
No opinion/Not qualified to judge	☐		

6. Compared to <u>gunshot</u> methodology for depopulation purposes, and considering the method as a whole, how does water-based foaming rank in your opinion?

	Worse	Same	Better
Overall method	☐	☐	☐
Logistics	☐	☐	☐
Animal welfare	☐	☐	☐
Speed	☐	☐	☐
Personnel safety	☐	☐	☐
Required resources	☐	☐	☐
Aesthetics (unpleasant to observe)	☐	☐	☐
No opinion/Not qualified to judge	☐		

7. Compared to <u>electrocution</u> methodology for depopulation purposes, and considering the method as a whole, how does water-based foaming rank in your opinion?

	Worse	Same	Better
Overall method	☐	☐	☐
Logistics	☐	☐	☐
Animal welfare	☐	☐	☐
Speed	☐	☐	☐
Personnel safety	☐	☐	☐
Required resources	☐	☐	☐
Aesthetics (unpleasant to observe)	☐	☐	☐
No opinion/Not qualified to judge	☐		

8. After seeing the foaming demonstration, in your opinion, how do the biosecurity protocols compare to other stationary indoor depopulation methodologies?

Worse	Same	Better	No opinion/Not qualified to judge
☐	☐	☐	☐

D. DIRECT AND INDIRECT STAFF HEALTH AND SAFETY

The foam used in this trial is approved by the Environmental Protection Agency (EPA) and is non-toxic and readily available and used by fire stations across the country. Despite the safe composition and characteristics of the foam, direct and indirect staff safety includes both acute practical risk as well as possible long-term effects.

1. How often do you think about previous euthanasia decisions made by yourself or colleagues?

☐ **Never** ☐ **Occasionally** ☐ **Frequently**

2. Would you consider the foaming method safe enough to eliminate risks of bodily harm of the staff/caretaker? ☐ **Yes** ☐ **No**

3. Would the foaming method be cause for concern regarding the mental fortitude of the staff/caretaker? ☐ **Yes** ☐ **No**

4. Did you personally find the foaming method discouraging, hard to observe or aversive to the extent that it would be challenging to repeatedly perform in the case of an emergency?

☐ **Yes** ☐ **No**

5. During or immediately after this trial, have you experienced repeated, disturbing memories, thoughts, or images of a stressful experience from the past?

☐ **Not at all** ☐ **A little bit** ☐ **Moderately** ☐ **Quite a bit** ☐ **Extremely**

6. During or immediately after this trial, have you felt upset as something reminded you of a stressful experience from the past?

☐ **Not at all** ☐ **A little bit** ☐ **Moderately** ☐ **Quite a bit** ☐ **Extremely**

7. During or immediately after this trial, have you avoided activities or situations because they reminded you of a stressful experience from the past?

☐ **Not at all** ☐ **A little bit** ☐ **Moderately** ☐ **Quite a bit** ☐ **Extremely**

8. During or immediately after this trial, have you felt irritable or had angry outbursts?

☐ **Not at all** ☐ **A little bit** ☐ **Moderately** ☐ **Quite a bit** ☐ **Extremely**

9. During or immediately after this trial, have you felt difficulty concentrating?

☐ **Not at all** ☐ **A little bit** ☐ **Moderately** ☐ **Quite a bit** ☐ **Extremely**

10. During or immediately after this trial, have you felt jumpy or easily startled?

☐ **Not at all** ☐ **A little bit** ☐ **Moderately** ☐ **Quite a bit** ☐ **Extremely**

Thank you very much for completing this questionnaire!
