# Supplementary material for: Swine industry stakeholders’ perception on the use of water-based foam as an emergency mass depopulation method
Source: PLoS One. 2023 Oct 20;18(10):e0290400. doi: 10.1371/journal.pone.0290400 (PMC10588842; doi:10.1371/journal.pone.0290400)
Supplement: S3 File — (PDF) [file pone.0290400.s003.pdf]

Default Question Block

Q1. What is your pre-assigned questionnaire number? It will be written on the piece of paper that had the link for this survey.

Q2.
Since you participated in the foaming depopulation trial a few weeks ago, have you experienced repeated, disturbing memories, thoughts, or images from the depopulation trial?

- Not at all
- A little bit
- Moderately
- Quite a bit
- Extremely

Q3.
Since you participated in the foaming depopulation trial a few weeks ago, have you experienced feeling very upset when something reminded you about the depopulation trial?

- Not at all
- A little bit
- Moderately
- Quite a bit

- Extremely

Q4.
Since you participated in the foaming depopulation trial a few weeks ago, have you avoided activities or situations because they reminded you about the foaming trial?

- Not at all
- A little bit
- Moderately
- Quite a bit
- Extremely

Q5.
Since you participated in the foaming depopulation trial a few weeks ago, have you felt irritable or had angry outbursts?

- Not at all
- A little bit
- Moderately
- Quite a bit
- Extremely

Q6.
Since you participated in the foaming depopulation trial a few weeks ago, have you had difficulty concentrating?

- Not at all
- A little bit
- Moderately

- Quite a bit
- Extremely

Q7.
Since you participated in the foaming depopulation trial a few weeks ago, have you felt jumpy or easily startled?

- Not at all
- A little bit
- Moderately
- Quite a bit
- Extremely
